# Poaching and human encroachment reverse recovery of African savannah elephants in south-east Angola despite 14 years of peace

**DOI:** 10.1371/journal.pone.0193469

**Published:** 2018-03-14

**Authors:** Scott Schlossberg, Michael J. Chase, Curtice R. Griffin

**Affiliations:** 1 Elephants Without Borders, Kasane, Botswana; 2 Department of Environmental Conservation, University of Massachusetts Amherst, Amherst, Massachusetts, United States of America; University of Tasmania, AUSTRALIA

## Abstract

With populations of African savannah elephants (*Loxodonta africana*) declining across the continent, assessing the status of individual elephant populations is important for conservation. Angola’s elephant population represents a key linkage between the larger populations of Namibia and Botswana. Elephants in Angola were decimated during the 1975–2002 Angolan civil war, but a 2005 survey showed that populations were recolonizing former habitats. Between 2005 and 2015, no research was permitted on elephants in Angola, but elsewhere in Africa many elephant populations experienced a poaching crisis. In 2015, we were able to resume elephant research in Angola. We used aerial surveys and satellite monitoring of collared elephants to determine the current status of elephant populations in Angola and to learn how human populations may be affecting elephant habitat usage. The aerial survey revealed a population of 3,395 ± SE of 797 elephants, but populations had declined 21% from the 2005 estimate. The high number of carcasses observed on the survey suggests that populations may have increased after the 2005 survey but were declining rapidly as of 2015. Satellite-collared elephants avoided areas <6 km from human indicators but preferred areas nearer humans at scales of 6–40 km, suggesting that humans may be displacing elephants from preferred habitats near rivers. Taken together, these results suggest that Angola’s elephant population is experiencing intense poaching and may be losing habitat to human settlements. Without action to conserve their populations, Angola’s elephants face an uncertain future.

## Introduction

Populations of African savannah elephants (*Loxodonta Africana*) and forest elephants (*Loxodonta cyclotis*) are declining in much of their ranges [1,2]. Recent elephant declines are primarily due to poaching [3], though habitat loss and human-elephant conflict (HEC) have also contributed [4,5]. Angola’s elephant populations have faced unique threats in recent decades. Prior to the 1970s, Angola was estimated to hold anywhere from 5,000 to 70,000 elephants [6]. Civil war wracked Angola from 1975 to 2002, resulting in widespread loss of human life and internal displacement of millions of people [7]. Anecdotal reports during the war suggested that wildlife, including elephants, were slaughtered on a mass scale. The rebel UNITA army killed elephants for meat and for their ivory, sales of which funded military operations [8]. An estimated 100,000 elephants were killed during the war, though some of this number may have originated outside Angola [9].

When the Angolan war ended in 2002, the status of elephants in the country was not known. In 2004–2005, Chase and Griffin [10] conducted the first systematic surveys for elephants in southeast Angola in 25 years. They found a small but apparently healthy and growing population, estimated at ~1,800 elephants as of November, 2005. Since 2004, at least 9 elephants tracked via satellite telemetry were observed dispersing from the larger populations of Namibia and Botswana into Angola (unpublished data). This led to optimism that Angola’s elephant population was recovering after the civil war [10]. Further surveys of elephants, however, were not permitted by the Angolan government from 2006 to 2015.

Despite the apparent success of elephant recovery in Angola, several factors led to growing concern about the status of Angola’s elephants during the period when surveys were not permitted. First, an outbreak of poaching has been decimating elephant populations across Africa since 2007 [2,3]. Second, though ivory sales and killing of elephants are illegal in Angola, those laws are little enforced. Angola joined the Convention on International Trade in Endangered Species (CITES) in 2013, but ivory sales were still commonplace there as of 2014 [11,12]. Additionally, CITES [13] considered the nation a likely location for international ivory trafficking. Finally, human populations in southeast Angola, where a large majority of the country’s elephants occur [14], have increased rapidly in recent years. The Cuando-Cubango province in southeast Angola was sparsely settled prior to the Civil War, but human populations there grew from 137,000 in 1995 to 534,000 in 2014 [15]. Consequently, HEC and habitat loss could potentially be having adverse effects on elephant populations [16].

**Fig 1:**
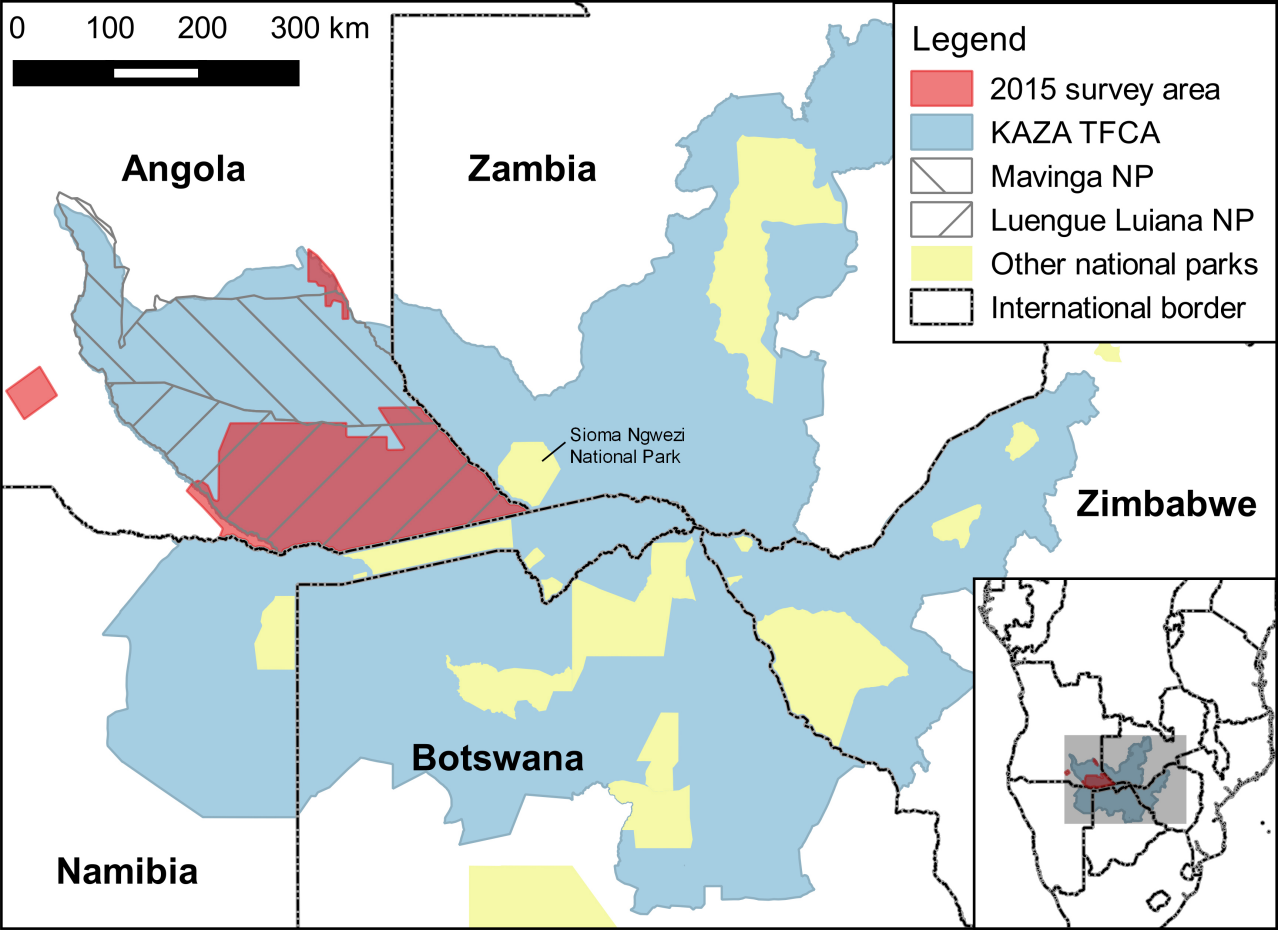
Study area for 2015 aerial surveys in Angola. Also shown are the Mavinga and Luengue Luiana National Parks in Angola, the Kavango-Zambezi Transfrontier Conservation Area (KAZA TFCA), and national parks in other KAZA TFCA countries.

Understanding the status of elephant populations in Angola has implications well beyond Angola’s borders. Satellite telemetry has shown that elephants in Angola move freely into Namibia and Zambia, sometimes travelling as far as Botswana and Zimbabwe (unpublished data). Such movements are important for connectivity of populations, maintaining genetic diversity, and allowing animals to seek out better habitats seasonally or in response to long-term environmental changes such as droughts [17,18]. To promote tourism and conserve natural resources regionally, Angola, Botswana, Namibia, Zambia, and Zimbabwe have proposed establishing the Kavango-Zambezi Transfrontier Conservation Area (KAZA TFCA) which would link protected areas and communal lands in the five countries. This initiative is potentially important for elephants because it would protect core elephant habitats and movement corridors between the countries [19]. For elephants, southeast Angola is a cornerstone of the KAZA TFCA, providing a key linkage between Namibia and Zambia (Fig 1). Failure of the southeast Angola population could seriously reduce connectivity between elephant subpopulations in the western half of the KAZA TFCA.

In 2015, the Angolan government granted Elephants Without Borders permission to resume research on elephant populations in southeast Angola. This allowed us to update the status of elephants in Angola for the first time since 2005. Here, we address three important questions about southeast Angola’s elephants: 1) How have elephant populations changed since 2005? 2) Has Africa’s recent poaching epidemic affected Angola? 3) How are growing human populations affecting habitat usage by elephants in Angola? We used aerial surveys and satellite telemetry of elephants to answer these questions.

## Methods

### Study area

Our study area was in the Cuando-Cubango province in southeast Angola (Fig 1). We conducted aerial surveys on a 43,459 km^2^ area where elephants had been observed in the past or were thought to occur based on consultation with local authorities. Most of the study area was in Luengue Luiana National Park (NP), with a small area in Mavinga NP. There were numerous subsistence agricultural settlements within the parks along the Kwando and Luiana Rivers (see Results). Our satellite telemetry of elephants incorporated a larger area of southeast Angola as well as adjacent sections of Zambia and Namibia.

Southeast Angola has a tropical climate, with a hot dry season (August-October), a hot wet season (November-April), and a cool dry season (May-July). Precipitation in the study area averages 828 mm/yr [10]. The topography is generally flat, and habitats include woodlands, shrublands, grasslands, and seasonally inundated floodplains. Four major rivers traverse the area, and human settlements are generally found along these rivers. Minefields left over from the civil war still remain in our study area and have injured humans, elephants, and other animals [10,20,21].

### Aerial surveys

We conducted aerial surveys for elephants as part of the Great Elephant Census (GEC), a 19-country survey of savannah elephants that took place in 2014–2015 [2]. In Angola, surveys took place from 12 October – 12 November, 2015, during Angola’s dry season. The study area was divided into 10 strata ranging in size from 1,672 to 14,094 km^2^ (S1 Fig). We used the sample transect method for our surveys. Accordingly, we surveyed a subset of each stratum with systematic transects, and we estimated population sizes of elephants by extrapolating from the survey area to the entire stratum. Sampling intensity, the proportion of a stratum actually censused, ranged from 4% to 19% (overall total: 8%) and increased with the expected density of elephants in the stratum (S1 Fig). Sampling intensity was controlled by the spacing between transects, which ranged from 2–10 km.

Roughly half of the surveys were conducted with a Cessna 182 single-engine aircraft; the remainder utilized a Cessna 206. Each plane had a crew of four: a pilot, a front recorder, and two rear observers. The pilot, front recorder, and left observer were the same in both aircraft; the right observer differed by aircraft. Samples were taken in strips demarcated with wands attached to the wing struts and calibrated on the ground to be 200 m wide on each side of the plane at the target altitude of 91 m. We determined the exact strip width on each side of the plane through repeated overflights of a runway marked at 10-m intervals [22]. During surveys, we recorded altitude at ~1-s intervals with a laser altimeter connected to a computer. Besides elephants, observers also counted elephant carcasses, other large and medium-sized mammals, and signs of human presence such as agricultural fields, huts, villages, poachers’ camps, and livestock. Poachers’ camps were identified by the presence of drying racks or weapons. Each observer had a camera that they could use to photograph larger elephant herds. We used these photographs to correct herd size estimates after flights were completed.

### Elephant collaring

Elephants Without Borders has been collaring elephants in Angola, Botswana, Namibia, and Zambia since 2001 to monitor elephant movements. To understand how human populations in Angola may be affecting elephants, we analyzed data from 8 collared elephants that were either collared in Angola or spent >100 days in Angola between 2010 and 2016. Methods used to tranquilize, collar, and awaken elephants are described in detail in Miller et al. [23]. All elephant darting was conducted by a wildlife veterinarian. The veterinarian darted the elephant with thianil hydrochloride to immobilize it. After the collar was attached, the veterinarian administered the antidote, naltroxine, and the elephant was typically alert and upright within 2–4 min. For each country where collaring was conducted, we had the permission of the relevant wildlife authority (Angola: National Director of Biodiversity, Ministry of the Environment; Botswana: Department of Wildlife and National Parks; and Namibia: Ministry of the Environment and Tourism). Collars were programmed to upload location data at intervals of 1–24 hr, depending on the collar’s battery life.

### Population change analysis

To estimate elephant population sizes on aerial surveys, we used a ratio estimator [24]. Accordingly, the density estimate for a stratum was the total number of animals observed within the survey strips divided by the total area sampled. To calculate the area sampled, we corrected the strip width of each transect for the mean altitude on that transect [25]. The population estimate, then, was the density estimate multiplied by the stratum area. We also used the ratio estimator variance to calculate the variance in the population estimate [24]. To estimate the population for the entire survey area, we summed the stratum population estimates. The variance of the population estimate for the entire survey was the sum of the stratum variances.

Because elephant carcasses remain visible for years after death, carcasses can be indicators of population growth rates of elephants [26]. Thus, for each stratum, we calculated the “carcass ratio” as $\frac{c}{\mathrm{e}+c}∙100\%$, where *c* is the number of carcasses observed and *e* is the number of live elephants observed. Carcass ratios >8% typically indicate a declining population, while lower ratios typically indicate a stable or growing population [26]. We also calculated the carcass ratio for the entire survey area. Because sampling intensity differed by stratum, we could not use the raw carcass counts to calculate the survey-wide carcass ratio. Rather, we estimated the total number of carcasses in the entire survey area with the ratio estimator, as described above. We divided this total by the estimated number of carcasses plus the estimated number of live elephants on all ten strata. We used the delta method to calculate the variance of this ratio.

Next, we sought to determine how elephant populations changed from 2005 to 2015. Chase and Griffin [9] reported results of three separate aerial surveys conducted in Angola in 2004 and 2005. We used their November, 2005 estimate as the basis for comparison because that survey, like the 2015 survey, was conducted in the dry season. The two remaining 2004–2005 surveys were wet-season surveys. The 2015 survey area was larger than the area surveyed in November, 2005 (S1 Fig). Thus, to make the 2005 and 2015 estimates comparable, we had to restrict the area sampled in 2015 to the area sampled in 2005. Accordingly, we used GIS software to limit the 2015 strata, transects, and observations to the 2005 survey area. We then calculated population estimates for the restricted strata as described above. We compared 2005 and 2015 population estimates with a two-sample *z* test.

We next determined how characteristics of elephant herds have changed over time. When threatened by poachers or human activity, elephants sometimes form large aggregations, with smaller herds merging together for safety [27,28]. Thus, changes in herd size or composition can be an indicator of threats to populations. We used *t*-tests to compare observed herd sizes between the 2005 and 2015 surveys. Because bull herds, composed solely of adult males, and breeding herds, composed of adult females and young of both sexes, generally differ in size, we compared herd sizes separately for each type of herd. Relative numbers of bull and breeding herds may also have changed over time in Angola. Previous research suggests that bulls may colonize new habitats before breeding herds because bulls tend to disperse more readily [29]. At the same time, bulls are preferentially killed by poachers because of their large tusks [30,31], so we might expect numbers of bulls to have decreased relative to breeding herds if poaching is a problem in Angola. To determine if sex ratios may have changed over time, we used Fisher’s exact test to compare the ratio of bull herds to breeding herds observed between 2005 and 2015.

### Measuring effects of human presence on elephants

We used two methods to examine effects of human presence on the distribution of elephants in southeast Angola. First, we examined correlations between signs of human activity and elephant presence on the 2015 aerial survey. Second, we used the locations of elephants tracked by satellite telemetry to determine how human presence affected elephants’ movements.

For the analysis of 2015 survey results, we divided each transect into 2.5-km segments to analyze correlations between elephant numbers and human presence. We restricted analyses to strata where elephants were observed; absence of elephants from other strata may be due to factors besides human activity. The 2.5-km distance was chosen to limit potential contamination due to unrecorded human activities in the strips between sample transects; longer distances would increase the probability that human activities in unsampled areas might affect our results. Fortunately, human indicators in Angola generally followed river valleys (see Results), and transects were generally perpendicular to rivers. Thus, locations of human activities should be approximated well by our survey data. For each segment, we calculated the number of elephants observed and the number of indicators of human presence: huts, villages, herds of livestock, farm fields, poaching or fishing camps, and logging sites or mills. Sample sizes for most indicators were too low to analyze individually. Thus, we summed observed numbers of all human indicators to calculate a single index of human activity for each transect segment. Obviously, different types of indicators may not have equal effects on elephants, but we were unable to analyze indicators individually.

Because 95% of the 670 transect segments analyzed had no elephants, the data were not well-suited for linear regression. Instead, we used mixed-effects logistic regression to determine how the summed number of human indicators affected whether elephants were present on transect segments. Because the amount of human activity varied by stratum, and environmental variables not measured likely differed by stratum as well, we included a random effect of stratum in the model. To account for the fact that segments at the end of transects were <2.5 km in length, we weighted observations in the regression by segment length.

We used resource selection functions (RSFs) as a second method to test effects of human presence on movements of collared elephants. RSFs are a broad class of models used to assess habitat preferences of animals by comparing used habitats with available or unused habitats [32]. We were interested in whether elephants avoid areas near human indicators and the spatial extent of any such avoidance. Thus, our RSFs compared used and available ranges to quantify habitat selection as a function of distance to human presence. Our analyses utilized data from six elephants collared in Angola in 2015 and two other elephants collared outside Angola that spent >100 days in Angola after 1 January 2010. We arbitrarily cut off our sample at 2010 because we sampled human presence via the 2015 aerial survey, and we wanted the elephant movement data to be relatively close in time. For each elephant, we divided the data into individual seasons, with the wet season running from 1 December through 30 April and the dry season from 1 May through 30 November. For each elephant and season, we then used the GPS locations to compute its 100% minimum convex polygon (MCP) home range. MCP home ranges overestimate actual areas used by animals [33,34] but are useful for identifying the area available for an animal to use in RSF models [35]. For each elephant and season, we then selected random points within the MCP home range to represent the area available to that elephant. The number of random points for each elephant and season was equal to the observed number of telemetry points for that elephant and season. We combined the used and random points in a single dataset and calculated each point’s distance to the nearest human feature in the 2015 aerial survey. Because the 2015 survey was a sample count, coverage of the study area was not complete. As discussed above, however, mapping the human indicators clearly showed that the indicators were clustered together along rivers (see Results). Consequently, the human signs recorded on the 2015 survey should be a good approximation of where humans are present in southeast Angola.

To compute the RSF, we used a hierarchical, logistic model in which the probability that a point was used, as opposed to random, was a function of distance to human indicators, elephant identity, and season [32]. Because we expected that the relationship between distance to human presence and elephant habitat usage would be non-linear, we used generalized additive mixed models (GAMM) to fit a smoothed curve for this relationship, with a logit link function. Our GAMM used penalized splines to account for expected non-linearity in the response of elephants to human activities. Models also included random intercept and smooth terms for each elephant/season to account for the correlated structure of the observations within seasons [see 36]. The random intercept reflected individual or seasonal variation in selectivity, and the random slope term reflected individual or seasonal variation in the shape of the curve relating habitat usage and distance to human activity. Treating elephant/season as a random effect was necessary because elephants may vary in their preference for or avoidance of human-dominated areas due to age, sex, and past experience with humans. Likewise, an individual elephant’s habitat preferences may vary from season to season depending on site-specific factors such as vegetation types, availability of water, or types of human activities nearby. We explored an alternate parameterization where observations were simply grouped by elephant instead of elephant and season, but the latter fit the data far better (unpublished data). We fitted the GAMM with the r package *mgcv* [36]. All smooths were thin-plate regression splines, and we used generalized cross-validation to determine the degrees of freedom for the smooths. We checked the final model for normality of residuals and heteroscedasticity. We note that the RSFs resulting from logistic regression have arbitrary units and should be interpreted as relative, not absolute, habitat preferences [37].

## Results

### Population change in elephants

**Fig 2:**
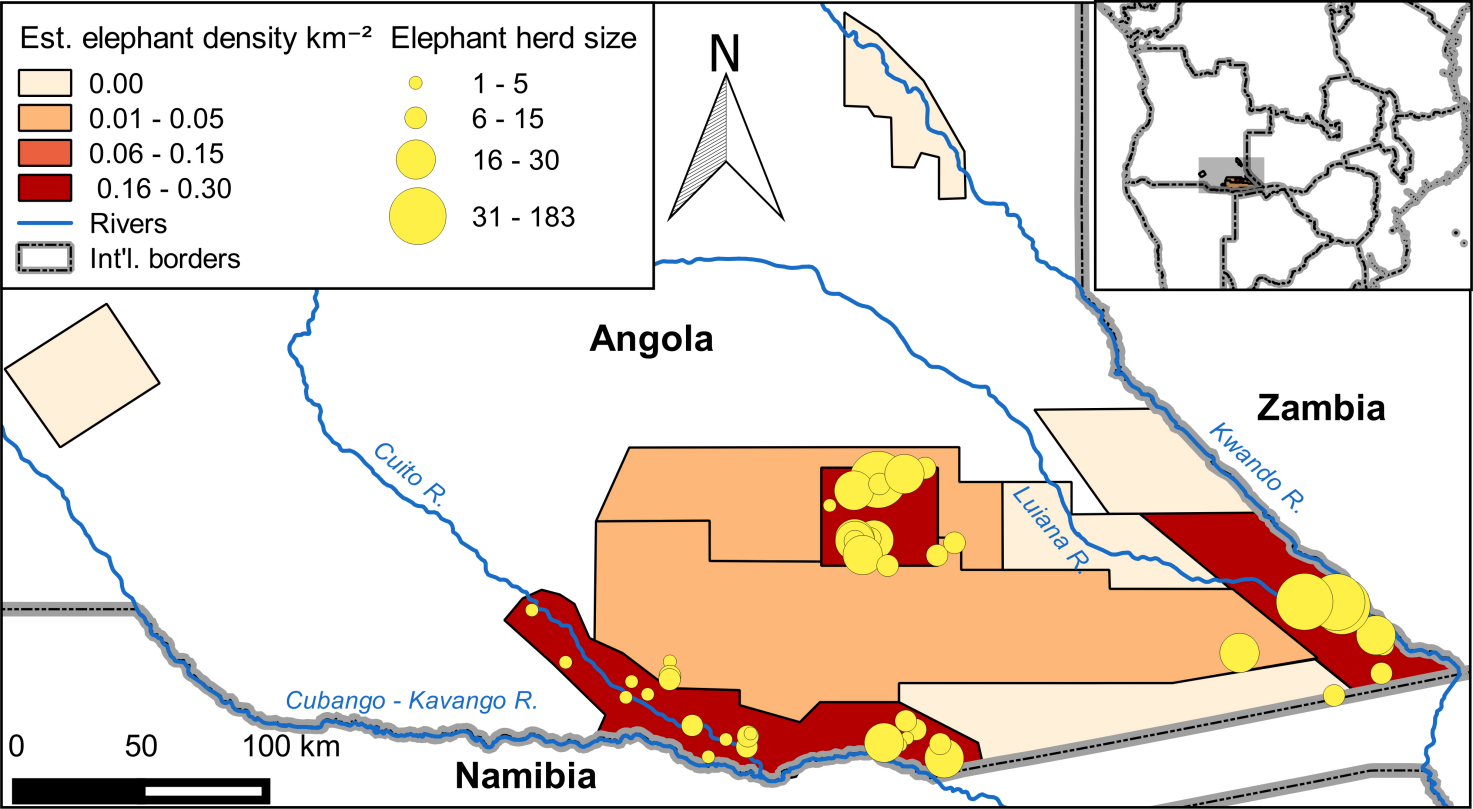
Estimated density of elephants and locations of observed herds in southeast Angola in 2015.

**Fig 3:**
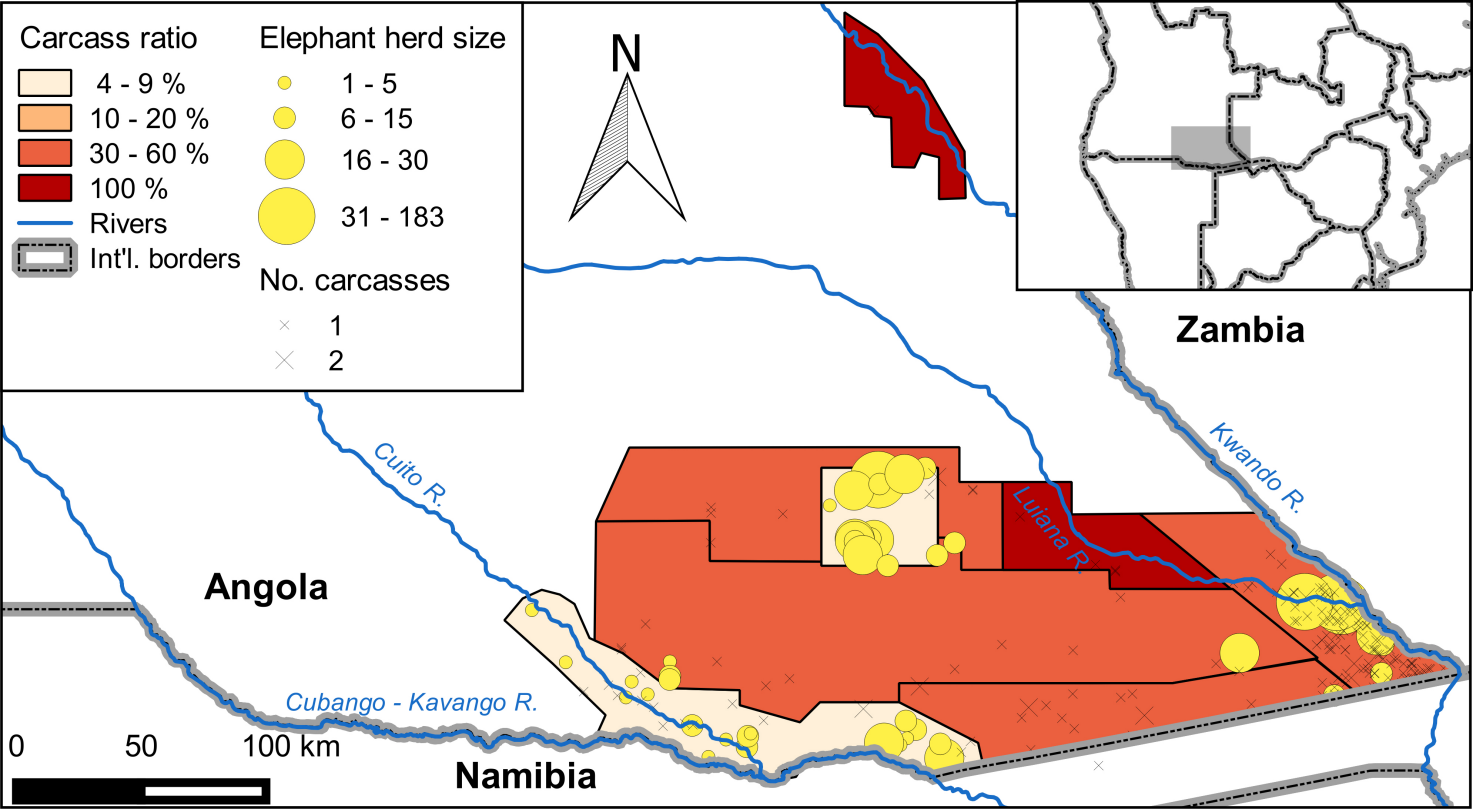
Estimated carcass ratios and locations of observed carcasses in southeast Angola in 2015.

Based on 2015 aerial surveys, we estimated a total population of 3,395 ± SE of 797 (95% CI: 1,778–5,012) elephants on our 43,459 km^2^ study area in southeast Angola (Fig 2). When we restricted the survey area to the area surveyed in 2005, we estimated a 2015 population of 1,437 ± 600 elephants. This was 21% decrease from the 2005 estimate of 1,827 ± 598 elephants, though the difference was not significant (*z* = 0.46, P = 0.65). Carcass ratios increased greatly between 2005 and 2015. On the 2005 survey, no elephant carcasses were observed. On the 2015 survey, the overall carcass ratio was 30 ± 3%, and carcass ratios were >8%, indicating a declining population, in seven of eight strata (Fig 3).

Mean sizes of elephant herds did not change significantly between 2005 and 2015 for either bull herds (2005: 3.4 ± SD of 4.5 elephants, 2015: 5.7 ± 5.2 elephants; *t*_23_ = 1.40, P = 0.17) or breeding herds (2005: 12.3 ± 8.3 elephants; 2015: 17.7 ± 15.8 elephants; *t*_26_ = 1.27, P = 0.22). In 2005, 16 of 42 herds observed (38%) were breeding herds. In 2015, 18 of 32 herds observed (56%) were breeding herds. This increase was not significant (Fisher’s exact test, P = 0.16).

### Effects of human presence

**Fig 4:**
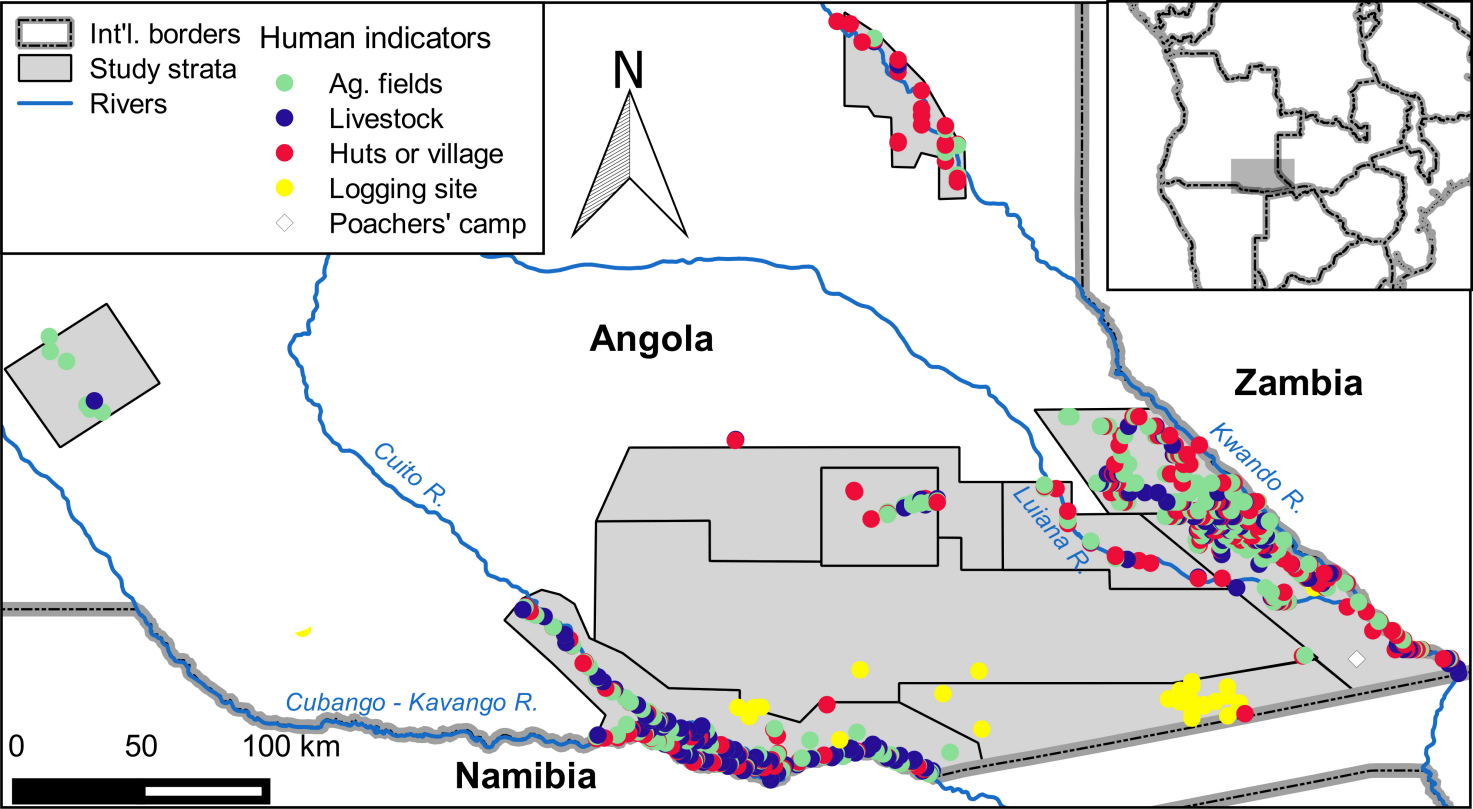
Locations where human indicators were observed in southeast Angola in 2015.

On the 2015 aerial survey of southeast Angola, we estimated a total of 18,697 indicators of human presence on the seven strata with elephants (Table 1; Fig 4). A logistic mixed-effects model showed a negative effect of human indicators on elephant presence (β = -0.72 ± 0.36, *z* = -1.99, P = 0.05). Elephants never occurred on any 2.5-km transect segment with >1 indicator of human presence (Fig 4).

**Table 1:** Estimated numbers of indicators of human presence in southeast Angola on the 2015 aerial survey. Estimates are only for strata where elephants were observed.

Indicator	Estimated number	SE
Agricultural fields	1,040	93
Fishing camps	11	7
Huts	955	122
Villages	180	39
Sheep / goats	133	119
Poachers’ camps	5	5
Logging sites	439	144
Cattle	15,934	1,611

**Fig 5:**
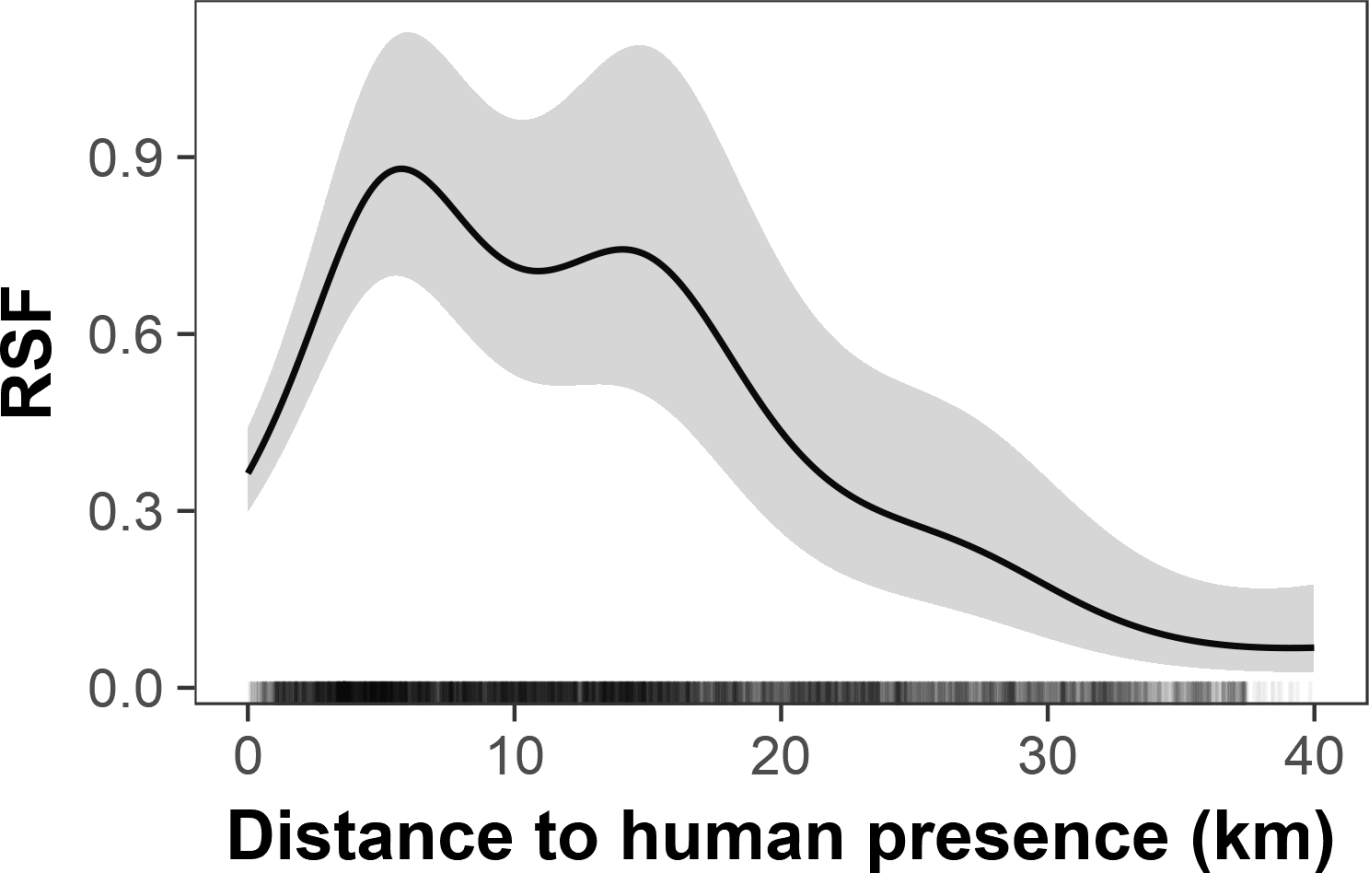
Predicted resource selection function (RSF) for elephants satellite-tracked in southeast Angola, 2010–2016. Shading indicates ± 1 SE. Tick marks on x-axis indicate density of data points from tracked elephants.

RSF analysis of elephant movements also showed avoidance of areas near human presence. We analyzed a total of 68,793 location fixes for the eight elephants used in the RSF study. Our GAMM showed a significant overall effect of distance to human presence on habitat usage (distance effect: P <0.0001). Accordingly, predicted elephant occurrence was greatest at 5.8 km from human presence and was lower both closer to and farther from that distance (Fig 5). Thus, elephants apparently avoided areas <5.8 km from human activity but were also averse to areas ≫5.8 km from human activity. The secondary peak in the RSF at ~15 km from human activities may be due to the fact that RSFs for some individual elephants had maxima at roughly this distance from human activities (S2 Fig).

## Discussion

### Elephant population status

Aerial surveys in southeast Angola indicated that elephant populations declined by 21%, equivalent to an exponential trend of *r* = -0.02, between 2005 and 2015. The 30% carcass ratio we observed, however, suggests a much more rapid decline. On the Great Elephant Census, two countries had carcass ratios similar to Angola’s; Tanzania had a 26% carcass ratio, and Mozambique’s was 32% [2]. Exponential growth rates for elephants in these two countries were -0.19 and -0.24, respectively, over the 4 years preceding the GEC. This suggests that our survey data may substantially underestimate the current rate of decline for elephants in Angola. Based on the observed carcass ratios, we believe that elephant populations in Angola continued to grow after the 2005 survey but then decreased much more rapidly than 2% per year in the years before our survey. The survey data likely underestimate recent declines because we did not measure the peak elephant population between 2005 and 2015. As further evidence for this hypothesis, on the 2015 survey, the carcass ratio for fresh carcasses, likely to have died within roughly one year before the survey, was 10%, suggesting a very high rate of recent mortality (unpublished data). This hypothesis is consistent with the observed increase in poaching that affected countries across Africa beginning around 2007 [2,3]. If our hypothesis about Angola’s elephants is correct, this country is in the midst of one of the worst poaching crises of any country in the savannah elephant’s range. Without corrective action, the nation’s elephant population is at serious risk of continued declines.

Elephant carcasses were widespread in our study area, with many observed in areas where we did not observe live elephants (Fig 3). These carcasses could represent locations occupied by elephants during the wet season, when they are free to disperse away from perennial water sources. During the 2004–2005 surveys of Angola, elephants were more widespread during the wet season than the dry season [10]. On the 2015 survey, the Likuwa Core stratum was notable because of its apparently healthy elephant population, with a carcass ratio of just 3.7%. This area also had fewer signs of human presence than other areas inhabited by elephants in Angola (Fig 4). Thus, the Likuwa Core area could make a useful focal point for future protection of elephant populations in this region.

Numerous landmines remain in southeast Angola following the civil war, and elephants have been killed by mines [21]. Three lines of evidence, however, suggest that poaching, not landmines, is the primary cause of the large number of carcasses observed on the 2015 survey. First, no carcasses were observed on the 2004–2005 elephant surveys [10]. If mines were killing elephants in large numbers, then this should have been apparent at that time. Second, Angola had a thriving domestic ivory market at least through 2014 [11,12]. Third, elephant populations across the Zambia border in Sioma Ngwezi NP have been decimated by poachers [2], as discussed further below. Taken together, these three lines of evidence suggest that illegal killing, not mines, is the primary cause of the high carcasses ratios in southeast Angola.

In Sioma Ngwezi NP, poaching reduced elephant populations from ~900 in 2004 to just 48 in 2015, and the carcass ratio was the highest recorded on the entire GEC [2]. Likewise, a 2014 survey in Botswana revealed increased poaching levels along the Kwando River, just across Namibia’s Caprivi strip from Angola [38]. These findings suggest that the plight of Angola’s elephants is part of a regional poaching problem. In some parts of Africa, poachers are known to cross borders to access elephant populations and then return to their home countries to evade law enforcement [39]. Consequently, cooperation between Angola and neighboring countries could aid in controlling poaching. The possibility exists that elephants may have moved into Angola from southwest Zambia to avoid poachers there, but survey and telemetry data are insufficient to determine how many elephants might have done so.

Losses of elephants in southeast Angola threaten the integrity of the proposed KAZA TFCA, which would protect elephant habitats and movement corridors from Namibia to Zimbabwe along the Cubango-Okavango and Zambezi Rivers and their tributaries. If elephants are unable to move safely across southeast Angola and southwest Zambia, the ability of the TFCA to link elephants across southern Africa will be greatly diminished. Veterinary fences along the Botswana-Namibia border in northwestern Botswana prevent elephants in Namibia from moving east through Botswana. Though parts of the Caprivi Strip are protected as national parks, other areas are heavily settled [40]. Consequently, excessive poaching and development in Angola could cause elephant populations in Namibia to become isolated from populations further to the east. Safeguarding Angola’s elephants and their habitats may be critical to the future success of the KAZA TFCA.

### Effects of human activities

A second major finding of our study was that human development is widespread in southeast Angola and may be limiting elephant distributions. We estimated that strata occupied by elephants in our survey area contain 1,040 agricultural fields, 15,000 livestock animals, and 180 villages (Table 1). Satellite tracking showed that elephants avoid areas within ~6 km of human indicators. As one moves from 6 to 40 km from human indicators, however, elephant habitat usage also declines, suggesting a preference for areas closer to humans. These seemingly contradictory findings may represent habitat selection by elephants on different scales. On scales of <6 km, elephants clearly avoid human-impacted areas. This finding was confirmed by our analysis of 2015 aerial survey data which showed elephant avoidance of human-impacted areas on scales of 2.5 km. On scales of 6–40 km, however, elephants appear to prefer areas nearer human indicators. This likely indicates a large-scale preference of elephants for floodplains or areas adjacent to floodplains. Human developments in southeast Angola are concentrated along major rivers (Fig 4). In fact, 77% of human indicators observed in the 2015 aerial survey were within 10 km of a perennial river (unpublished data). Elephants in Angola may, like humans, also prefer areas along rivers, either because of the availability of favored foods or for access to water [41]. Thus, humans may be displacing elephants from preferred habitats along rivers in southeast Angola. The preferences of both elephants and humans for riverine habitats could expose elephants to HEC, though the extent of such conflict has not been evaluated in Angola. The potential for humans to displace elephants may increase if Angola follows through on ambitious plans to develop large-scale irrigation projects along the Cubango-Okavango River [42].

Although elephant populations have declined in southeast Angola, we did not observe significant changes in herd sizes or herd composition between 2005 and 2015. There was a non-significant trend towards breeding herds being more common in 2015, which is consistent with known behaviors of elephants and poachers. Bull elephants are more likely than females to undertake long-distance movements [29], so bulls likely made up most of the early dispersers to southeast Angola after the civil war. Family groups with females and young, which tend to be more site faithful, therefore, arrived after bulls. Thus, the increase in the relative number of family herds may simply be due to dispersing family groups finally reaching Angola. At the same time, numbers of bulls may have decreased relative to females and young because bulls are preferentially poached for their tusks [30,31].

We did not observe any significant changes in herd size between 2005 and 2015, but herd sizes were highly variable in both surveys, limiting the power of our tests. On the 2015 survey, however, we observed a single herd of >450 elephants, the majority of which were outside of the survey strip. This was the largest single herd observed on the entire 18-country GEC (unpublished data). Elsewhere in Africa, elephants have been known to form such large aggregations in response to persecution by humans [27,28]. The presence of this large herd may be another sign of poaching and harassment of elephants by humans in Angola.

In conclusion, the elephant population in southeast Angola appears to be in a precarious position, with intensive poaching reducing populations and encroaching human settlements affecting elephant distributions and potentially reducing elephants’ access to preferred floodplain and riparian habitats. Numerous studies have shown that war has negative effects on wildlife populations [10,43]. Our study shows that ending war is not necessarily sufficient for the long-term recovery of wildlife populations. Active protection of wildlife is also needed. Angola is one of the poorest countries in Africa, so resources for fighting poaching or conserving elephant habitats are likely to be highly limited. Since the 2015 survey, however, the Angolan government has taken several steps to protect elephants. The government has submitted a national ivory action plan to CITES, enacted new legislation to make the sale of live or dead wildlife a crime, and stepped up seizures of elephant ivory [44,45]. With over 3,000 elephants as of late 2015, Angola may still have time to reverse the ongoing decline of elephants in Angola and conserve this internationally important population.

## Supporting information

S1 Fig Study design for the 2015 aerial survey in southeast Angola. Coverage is the percentage of the stratum that was sampled on the survey.
(TIFF)

S2 Fig Estimated resource selection functions (RSFs) for individual elephants and seasons. Each line indicates an RSF for an individual elephant and season.
(TIFF)

S1 Data Aerial survey data used to estimate elephant population sizes and numbers of human indicators in study strata. (XLSX)

S2 Data Aerial survey data used to estimate effects of human indicators on presence/absence of elephants. (XLSX)

S3 Data Elephant tracking data and availability data (random points) used to estimate resource selection functions for distance to human indicators. (XLSX)

## References

[1] Maisels F, Strindberg S, Blake S, Wittemyer G, Hart J, Williamson EA, et al Devastating decline of forest elephants in Central Africa. PLoS ONE. 2013;8: e59469 doi: 10.1371/journal.pone.0059469 23469289 10.1371/journal.pone.0059469 PMC3587600

[2] Chase MJ, Schlossberg S, Griffin CR, Bouché PJC, Djene SW, Elkan PW, et al Continent-wide survey reveals massive decline in African savannah elephants. PeerJ. 2016;4: e2354 doi: 10.7717/peerj.2354 27635327 10.7717/peerj.2354 PMC5012305

[3] Wittemyer G, Northrup JM, Blanc J, Douglas-Hamilton I, Omondi P, Burnham KP. Illegal killing for ivory drives global decline in African elephants. Proc Natl Acad Sci. 2014;111: 13117–13121. doi: 10.1073/pnas.1403984111 25136107 10.1073/pnas.1403984111 PMC4246956

[4] Newmark WD. Isolation of African protected areas. Front Ecol Environ. 2008;6: 321–328. doi: 10.1890/070003

[5] Mariki SB, Svarstad H, Benjaminsen TA. Elephants over the cliff: explaining wildlife killings in Tanzania. Land Use Policy. 2015;44: 19–30. doi: 10.1016/j.landusepol.2014.10.018

[6] Anstey S. Angola: elephants, people and conservation, a preliminary assessment of the status and conservation of elephants in Angola. Harare, Zimbabwe: IUCN Regional Office for Southern Africa; 1993.

[7] de Oliveira RS. Magnificent and beggar land: Angola since the civil war. Oxford: Oxford University Press; 2015.

[8] Breytenbach J. The plunderers. Johannesburg, South Africa: Covos Day; 2001.

[9] Kumleben ME. Commission of inquiry into the alleged smuggling of and illegal trade in ivory and rhinoceros horn in South Africa. Durban, South Africa: Report to the State President of the Republic of South Africa; 1996.

[10] Chase MJ, Griffin CR. Elephants of south-east Angola in war and peace: their decline, re-colonization and recent status. Afr J Ecol. 2011;49: 353–361.

[11] Vigne L, Martin E. Findings on the flourishing ivory trade in Angola’s capital, Luanda. TRAFFIC Bull. 2014;26: 44–46.

[12] Svensson MS, Bersacola E, Bearder SK, Nijman V, Mills M. Open sale of elephant ivory in Luanda, Angola. Oryx. 2014;48: 13–14. doi: 10.1017/S0030605313001439

[13] CITES. ETIS report of TRAFFIC. COP16 Doc. 43.2.2 (rev.1). Bangkok, Thailand: CITES; 2013.

[14] Thouless CR, Dublin HT, Blanc JJ, Skinner DP, Daniel TE, Taylor RD, et al African elephant status report 2016: an update from the African Elephant Database. Gland, Switzerland: IUCN; 2016.

[15] Ceita C. Resultados definitivos do recenseamento geral da populacao e da habitacao de Angola 2014. Luanda, Angola: Gabinete Central do Censo; 2016.

[16] Wallenfang J, Finckh M, Oldeland J, Revermann R. Impact of shifting cultivation on dense tropical woodlands in southeast Angola. Trop Conserv Sci. 2015;8: 863–892.

[17] Epps CW, Wasser SK, Keim JL, Mutayoba BM, Brashares JS. Quantifying past and present connectivity illuminates a rapidly changing landscape for the African elephant. Mol Ecol. 2013;22: 1574–1588. doi: 10.1111/mec.12198 23398457 10.1111/mec.12198

[18] Roever CL, van Aarde RJ, Chase MJ. Incorporating mortality into habitat selection to identify secure and risky habitats for savannah elephants. Biol Conserv. 2013;164: 98–106. doi: 10.1016/j.biocon.2013.04.006

[19] Hanks J, Myburgh W. The evolution and progression of transfrontier conservation areas in the Southern African development community In: van der Duim R, Lamers M, van Wijk J, editors. Institutional Arrangements for Conservation, Development and Tourism in Eastern and Southern Africa. New York: Springer; 2015 pp. 157–179.

[20] Arcand J-L, Rodella-Boitreaud A-S, Rieger M. The impact of land mines on child health: evidence from Angola. Econ Dev Cult Change. 2015;63: 249–279. doi: 10.1086/679069

[21] Berhe AA. The contribution of landmines to land degradation. Land Degrad Dev. 2007;18: 1–15. doi: 10.1002/ldr.754

[22] Frederick H, Moyer D, Plumptre AJ. Aerial procedures manual, version 1.0. Wildlife Conservation Society; 2010.

[23] Miller LJ, Chase MJ, Hacker CE. A comparison of walking rates between wild and zoo African elephants. J Appl Anim Welf Sci. 2016;19: 271–279. doi: 10.1080/10888705.2015.1137755 26963741 10.1080/10888705.2015.1137755

[24] Jolly GM. Sampling methods for aerial censuses of wildlife populations. East Afr Agric For J. 1969;34: 46–49. doi: 10.1080/00128325.1969.11662347

[25] Norton-Griffiths M. Counting animals. Nairobi, Kenya: African Wildlife Foundation; 1978.

[26] Douglas-Hamilton I, Burrill A. Using elephant carcass ratios to determine population trends African Wildlife: Research and Management. Paris, France: International Council of Scientific Unions; 1991 pp. 98–105.

[27] Eltringham SK, Malpas RC. The decline in elephant numbers in Rwenzori and Kabalega Falls National Parks, Uganda. Afr J Ecol. 1980;18: 73–86. doi: 10.1111/j.1365-2028.1980.tb00271.x

[28] Poole J. The African elephant In: Kangwana K, editor. Studying elephants. Nairobi, Kenya: African Wildlife Foundation; 1996 pp. 1–8.

[29] Osborn FV, Parker GE. Linking two elephant refuges with a corridor in the communal lands of Zimbabwe. Afr J Ecol. 2003;41: 68–74. doi: 10.1046/j.1365-2028.2003.00413.x

[30] Poole JH, Thomsen JB. Elephant are not beetles: implications of the ivory trade for the survival of the African elephant. Oryx. 1989;23: 188–198. doi: 10.1017/S0030605300023012

[31] Wittemyer G. The elephant population of Samburu and Buffalo Springs National Reserves, Kenya. Afr J Ecol. 2001;39: 357–365. doi: 10.1046/j.1365-2028.2001.00324.x

[32] Manly BFJ, Mcdonald LL, Thomas DL, Mcdonald TL, Erickson WP. Resource selection by animals: statistical design and analysis for field studies. Second edition. 2002.

[33] Burgman MA, Fox JC. Bias in species range estimates from minimum convex polygons: implications for conservation and options for improved planning. Anim Conserv Forum. 2003;6: 19–28. doi: 10.1017/S1367943003003044

[34] Downs JA, Horner MW. Effects of point pattern shape on home-range estimates. J Wildl Manag. 2008;72: 1813–1818. doi: 10.2193/2007-454

[35] Thomas DL, Taylor EJ. Study designs and tests for comparing resource use and availability II. J Wildl Manag. 2006;70: 324–336. doi: 10.2193/0022-541X(2006)70[324:SDATFC]2.0.CO;2

[36] Wood S. Generalized additive models: an introduction with r. CRC press; 2006.

[37] Johnson CJ, Nielsen SE, Merrill EH, Mcdonald TL, Boyce MS. Resource selection functions based on use–availability data: theoretical motivation and evaluation methods. J Wildl Manag. 2006;70: 347–357. doi: 10.2193/0022-541X(2006)70[347:RSFBOU]2.0.CO;2

[38] Chase M, Schlossberg S, Landen K, Sutcliffe R, Seonyatseng E, Keitsile A, et al Dry season aerial survey of elephants and wildlife in northern Botswana, July-October 2014. Kasane, Botswana: Elephants Without Borders; 2015.

[39] Poulsen JR, Koerner SE, Moore S, Medjibe VP, Blake S, Clark CJ, et al Poaching empties critical central African wilderness of forest elephants. Curr Biol. 2017;27: R134–R135. doi: 10.1016/j.cub.2017.01.023 28222286 10.1016/j.cub.2017.01.023

[40] Chase MJ, Griffin CR. Elephants caught in the middle: impacts of war, fences and people on elephant distribution and abundance in the Caprivi Strip, Namibia. Afr J Ecol. 2009;47: 223–233.

[41] de Knegt HJ, van Langevelde F, Skidmore AK, Delsink A, Slotow R, Henley S, et al The spatial scaling of habitat selection by African elephants. J Anim Ecol. 2011;80: 270–281. doi: 10.1111/j.1365-2656.2010.01764.x 21054380 10.1111/j.1365-2656.2010.01764.x

[42] OKACOM (The Permanent Okavango River Basin Water Commission). Synthesis report: Cubango-Okavango River Basin water audit (CORBWA) project. Rome: Food and Agriculture Organization of the United Nations; 2014.

[43] Dudley JP, Ginsberg JR, Plumptre AJ, Hart JA, Campos LC. Effects of war and civil strife on wildlife and wildlife habitats. Conserv Biol. 2002;16: 319–329. doi: 10.1046/j.1523-1739.2002.00306.x

[44] Vieira I. Angola criminalises poaching. In: Africa Review [Internet]. 2 Jun 2017 [cited 17 Jan 2018]. Available: http://www.africareview.com/news/Angola-criminalises-poaching—/979180-3967138-format-xhtml-hinw4qz/index.html

[45] Hungerford E. Government bans trade of ivory in Angola. In: The Independent [Internet]. 21 Mar 2016 [cited 17 Jan 2018]. Available: http://www.independent.co.uk/voices/campaigns/GiantsClub/government-bans-trade-of-ivory-in-angola-a6944486.html

