# Supplementary figures and images for: Poaching and human encroachment reverse recovery of African savannah elephants in south-east Angola despite 14 years of peace
Source: PLoS One. 2018 Mar 14;13(3):e0193469. doi: 10.1371/journal.pone.0193469 (PMC5851583; doi:10.1371/journal.pone.0193469)

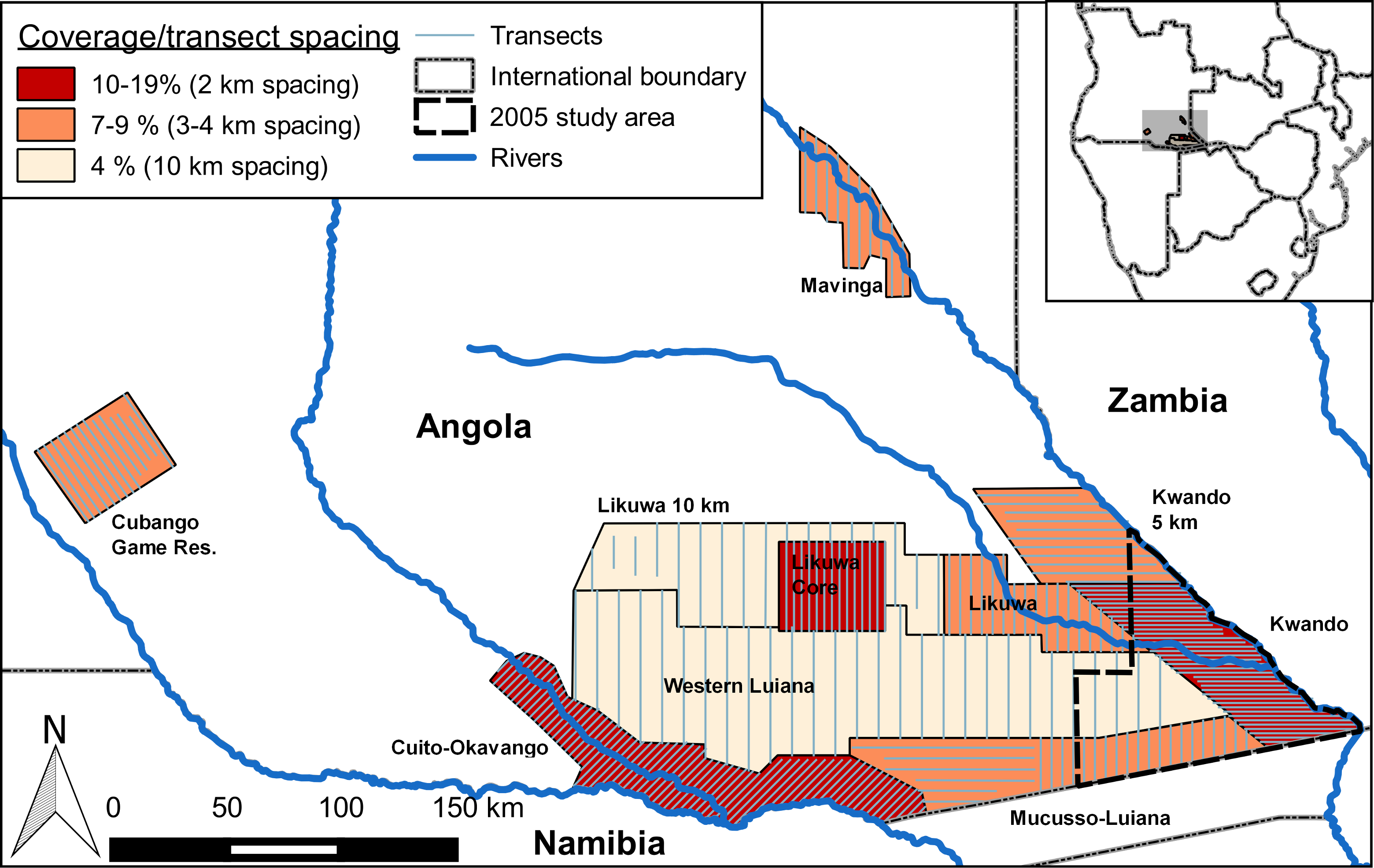

Supplement: S1 Fig — Coverage is the percentage of the stratum that was sampled on the survey. (TIFF) [file pone.0193469.s001.tiff]

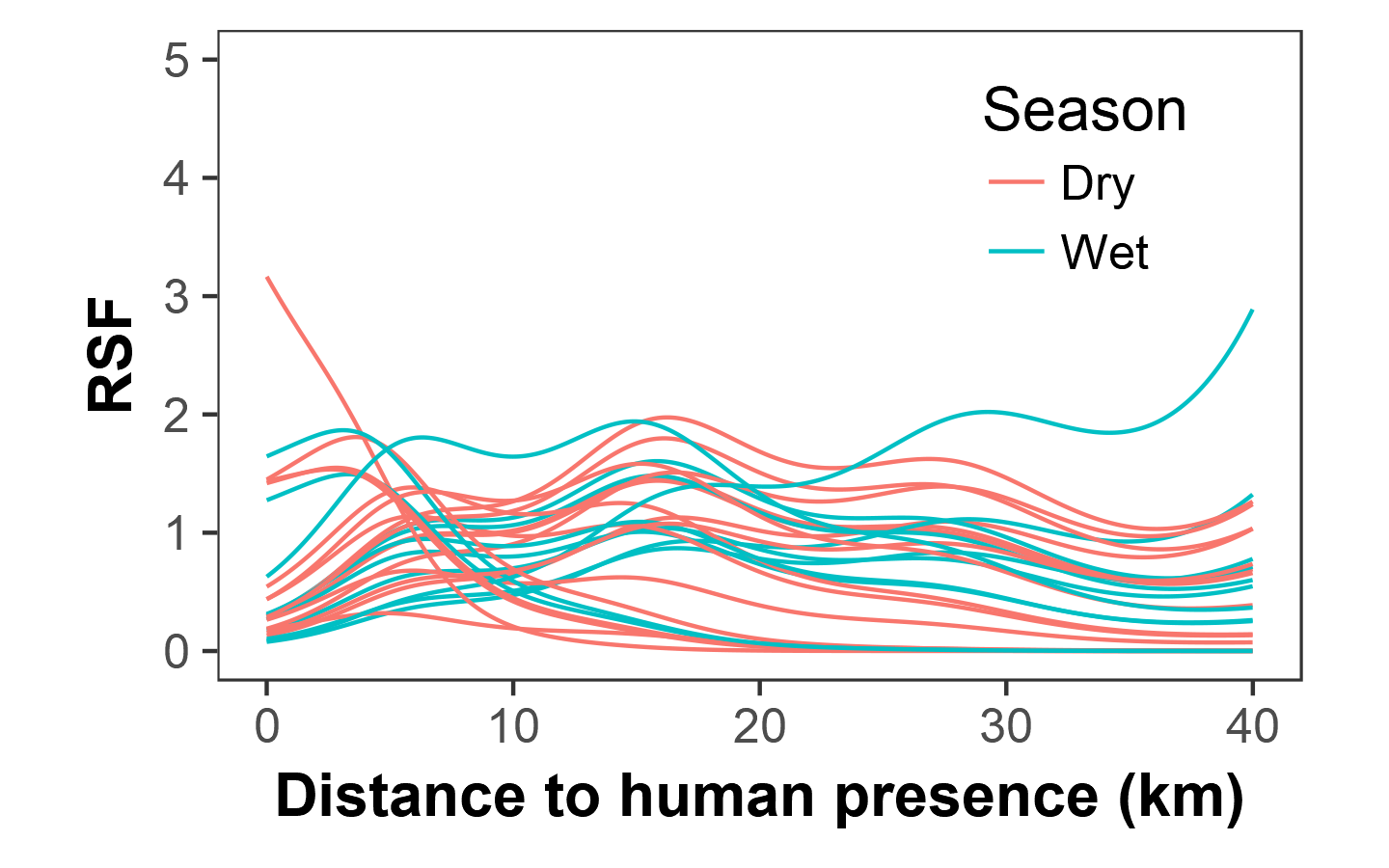

Supplement: S2 Fig — Each line indicates an RSF for an individual elephant and season. (TIFF) [file pone.0193469.s002.tiff]
